# Psychological resilience trajectories after the COVID-19 pandemic: the role of lifestyle and psychosocial factors in a cohort at increased alzheimer’s disease risk

**DOI:** 10.1007/s00127-025-02937-w

**Published:** 2025-06-23

**Authors:** Israel Contador, Müge Akinci, Eleni Palpatzis, Pablo Aguilar-Domínguez, Carme Deulofeu, Sherezade Fuentes-Julian, Karine Fauria, Carolina Minguillón, Oriol Grau-Rivera, Gonzalo Sánchez-Benavides, Eider M. Arenaza-Urquijo

**Affiliations:** 1 Department of Basic Psychology, Psychobiology and Methodology of Behavioural Science, University of Salamanca, Salamanca, Spain; 2 Hospital del Mar’ Research Institute, Barcelona, Spain; 3 Barcelonaβeta Brain Research Center (BBRC), Pasqual Maragall Foundation, Barcelona, Spain; 4 Barcelona Institute for Global Health (ISGlobal), Barcelona, Spain; 5 University of Pompeu Fabra (UPF), Barcelona, Spain; 6 Sant Pau Memory Unit, Department of Neurology, Hospital de la Santa Creu i Sant Pau, Biomedical Research Institute Sant Pau (IIBSant Pau), Universitat Autónoma de Barcelona, Barcelona, Spain; 7 Centro de Investigación Biomédica en Red de Fragilidad y Envejecimiento Saludable, Instituto de Salud Carlos III, Madrid, Spain; 8 Servei de Neurología, Hospital del Mar, Barcelona, Spain

**Keywords:** Resilience, COVID-19, Mental health, Depression, Alzheimer’s disease

## Abstract

**Purpose:**

This longitudinal cohort study evaluates whether lifestyle and psychosocial factors are associated with psychological resilience at two time points of COVID-19 pandemic. Moreover, we investigated the mediating role of perceived stress on these associations.

**Methods:**

A total of 677 cognitively unimpaired (CU) older adults at increased risk of Alzheimer’s disease (AD) completed the Hospital Anxiety and Depression Scale (HADS). Based on the Reliable Change Index (RCI), HADS trajectories were defined at two intervals: (1) pre-pandemic-confinement (follow-up = 2,28 ± 0,84 years); (2) confinement-post-confinement (follow-up = 1,49 ± 0,12 years). Then, 4 trajectory groups were defined: Psychological Resilience (*n* = 448, stable or improve at both intervals), Descending (*n* = 84, stable/improve [interval 1]-worsen [interval 2]), Recovery (*n* = 59, worsening [interval 1], improvement [interval 2] ) and Non-resilient (*n* = 86, worsening at both intervals). Logistic regression models (LRM) were applied considering lifestyle (physical and leisure activities, sleep) and psychosocial factors (social relationships and emotional support) as predictors of psychological resilience trajectory (i.e., outcome) at both intervals. Finally, mediation analyses were carried out to test the effect of perceived stress on the relationships between the predictive factors and psychological resilience.

**Results:**

Our finding showed that most participants followed a psychological resilient trajectory (66,1%). LRMs indicated that higher physical activity level, a greater number of social interactions and longer sleep duration were significantly associated with a psychological resilience trajectory both at confinement and at the 1.5 years follow-up. Lastly, the mediation analyses suggested that these factors influence psychological resilience through the mitigation of perceived stress.

**Conclusion:**

These findings underscore the role of physical activity, social interactions and sleep quality to strengthen individuals’ capacity to cope with stress during prolonged crisis such as the COVID-19 pandemic. These lifestyle and psychological factors may be valuable targets for public health strategies aimed to prevent mental health problems.

## Introduction

The COVID-19 pandemic, recognized as an international public health emergency [1], forced the governments to implement restrictive policies (i.e., lockdowns and social distance), that had a large impact on health and well-being of the populations [2, 3]. Particularly, the pandemic affected older adults, with over 95% of associated deaths involving individuals over the age of 50 years [4]. While mitigation strategies such as lockdowns increased social isolation in the older population [5, 6], their impact on mental health has been highly heterogeneous, with individuals showing trajectories ranging from resilience to psychological distress [7, 8]. Therefore, identifying modifiable factors associated with more favourable psychological trajectories in vulnerable populations can promote the development of targeted interventions and preparedness for future pandemics.

Research on psychological trajectories in older adults is of particular interest, as behavioural and psychological symptoms (e.g., anxiety and depression) have been associated with increased risk of cognitive impairment and Alzheimer’s disease (AD) dementia [9, 10]. In fact, anxiety and depression may be manifestations of AD pathophysiology (amyloid-beta and tau burden) at the preclinical stage of the disease, prior to the onset of cognitive impairment [11–13]. Moreover, these symptoms have been associated with progression to dementia in individuals with cognitive impairment [14, 15].

Although studies across different countries have focused on psychological trajectories during and after the COVID-19 in the general population [7, 16–18], fewer have addressed psychological changes in older adults. The existing evidence shows that a large proportion of both the general population and older adults remains psychologically stable or resilient over time [19–26]. Despite this, there has not been a focus on cohorts enriched for AD risk. This is relevant as previous research has shown an association of AD pathologies and worsening mental health in cognitively unimpaired (CU) adults at risk for AD during the COVID-19 confinement [27]. Therefore, it is essential to understand the modifiable factors that allow older adults, especially those at risk for AD, to remain resilient to mental health problems derived from prolonged stressful situations such as pandemics.

During the COVID-19 pandemic, several sociodemographics factors were associated with worse mental health, including younger age, being a woman, lower education, living without a partner, unemployment and lower income [8, 16, 28]. Further, pre-existing mental disorders, personality traits (neuroticism), negative feelings/emotions (loneliness, hopelessness) and inadequate coping strategies (i.e., behavioural disengagement, substance abuse) were shown to increase mental distress [29–33]. Conversely, lifestyle and psychosocial factors such as physical activity [34], better sleep quality [28], social support [35], and psychological resources (i.e., self-efficacy) [22] were associated with better psychological trajectories in different populations during lockdowns. Finally, previous research has shown that perceived stress during the COVID-19 pandemic may mediate the association between specific lifestyle (i.e., physical activity), psychosocial factors (i.e., personality traits, social support) and mental health problems [36–39]. These studies suggest that perceived stress acts as a pathway through which these factors exert their influence on psychological distress during the COVID-19 pandemic.

The current study aims to address several gaps in the existing literature. First, while studies are often focused on risk factors associated with distress and mental health problems during the pandemic [26, 40, 41], factors promoting psychological resilience on individuals over 50–55 years of age have received less attention [42, 43]. Additionally, most of the studies on mental health problems are cross-sectional and, therefore, do not consider pre-confinement status measures or long-term follow-ups [7]. This is important, as pandemics are dynamic events and individuals’ reactions vary over time [44–46]. Lastly, previous studies have often applied individual-centred statistical techniques (i.e., latent class growth analysis) to identify psychological trajectories (i.e., depression). However, these approaches, which rely on probabilities to assign the individual’s class, do not guarantee accurate class assignment or the reliability of psychological changes [47, 48]. Therefore, investigating psychological trajectories in older adults, through a longitudinal study design, with a focus on clinically significant changes appears crucial.

The objectives of the current study were twofold. First, we aimed to investigate the association of different lifestyle and psychosocial factors with psychological resilience trajectories at two time points of the pandemic (during confinement and 1.5 after) in an AD risk-enriched cohort. Second, we investigated whether perceived stress mediates the relationship between these lifestyle/psychosocial factors and psychological trajectories.

## Methods

### Participants

**Fig. 1:**
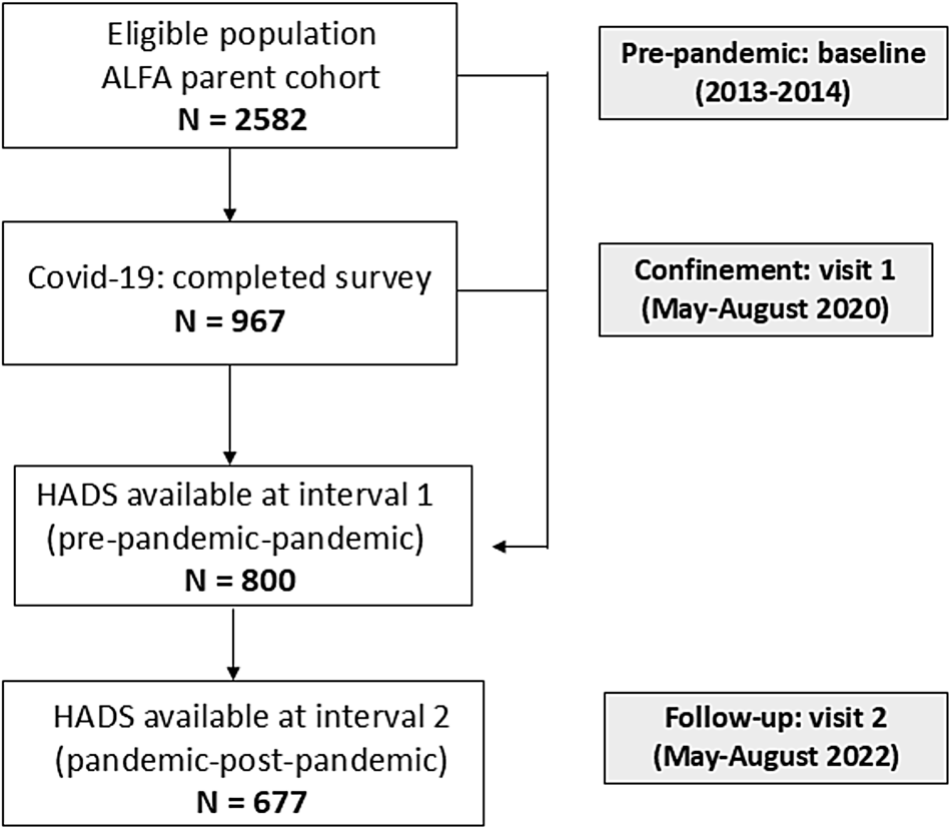
Flow chart of the study baseline assessment of follow-up

Participants of the present study were part of the ALFA (ALzheimer and FAmilies) parent cohort, recruited between 2013 and 2014 (baseline visit). The ALFA cohort includes 2743 middle-aged (45–74 years) CU individuals, the majority with a family history of sporadic AD [49]. An invitation to participate in the current study was sent (via an online link) to 2582 participants of the ALFA parent cohort (i.e., eligible participants) during de-escalation phases of the COVID-19 confinement (May 8, 2020). Of them, 967 completed the COVID questionnaire between May-August 2020 (visit 1). Among these individuals, 800 had the Hospital Anxiety and Depression Scale (HADS) available at visit 1 (i.e., pre-pandemic to confinement) and 667 at second visit or follow-up (i.e., 1.5 years after confinement) (Fig. 1). The average time lapses were 2,28 ± 0,84 years (follow-up 1) and 1,49 ± 0,12 years (follow-up 2), respectively. All participants signed an informed consent for the present study using an online form.

### Human participant protection statement

The COVID-19 protocol (CovidImpact_BBRC2020) was approved on March 16, 2020 (2020/9255) according to the ethical standards established in the Declaration of Helsinki (1964) and its later amendments.

### Measures

**Fig. 2:**
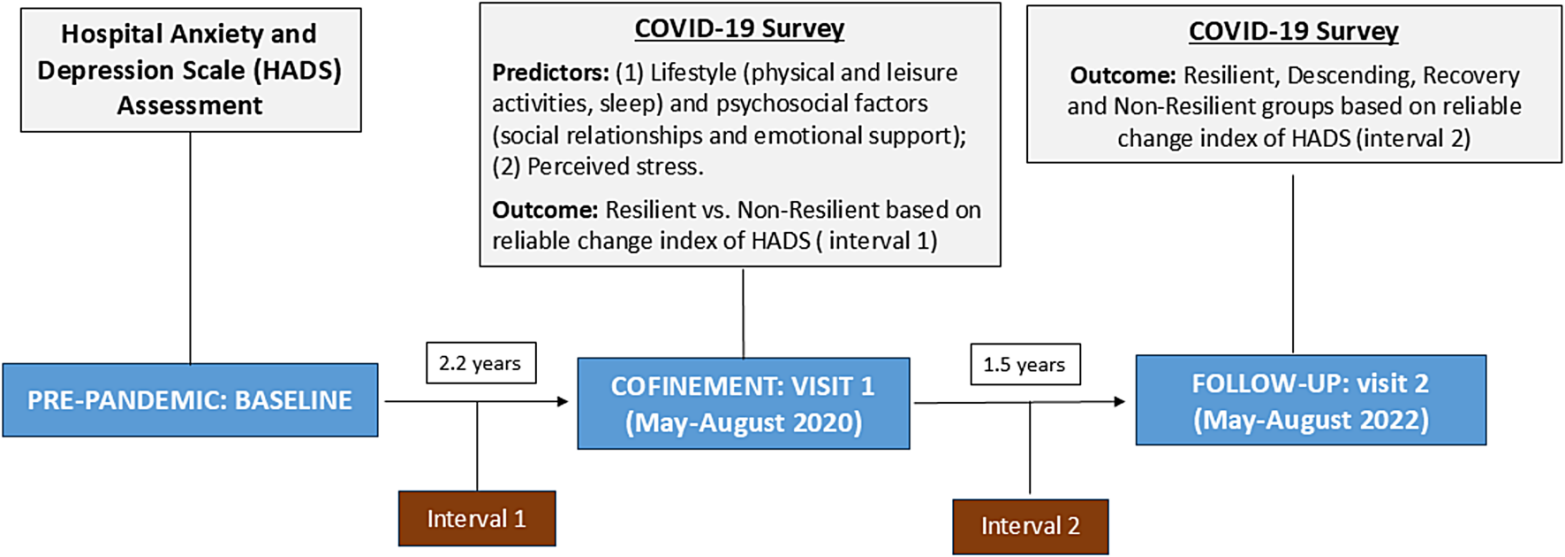
Design of the study: timeline and main variables

Figure 2 depicts the design of the study, main predictive factors and outcomes at different intervals.

*Anxiety and depression.* The HADS is composed of the 7-item anxiety and the 7-item depression subscales (14 items) that measure symptoms of anxiety/depression. In each subscale, the items are scored from 0 to 3, generating a total score between 0 and 21. Higher scores indicate a greater level of anxiety or depression [50].

### Outcomes

*Definition of resilient vs. non-resilient participants*. The classification was established based on the Reliable Change Index (RCI) in the HADS scale at different follow-ups (pre-pandemic to confinement and confinement to post-confinement). RCIs were calculated using Hsu’s method [51], which takes into account the effect of regression to the mean (i.e., increase of measurement errors associated with extreme scores). Accordingly, RCI = ((X2-M2) - (r_xy_* (X1-M1))/SEP, with X1 = observed pre-test score, X2 = observed post-test score, M1 = group mean pre-test score and M2 = group mean post-test score, and the Standard Error of Prediction (SEP) = SD*(1 - r^2^)^1⁄2^. SD is the Standard Deviation of the HADS at T1 and r = test-retest reliability coefficient. RCI values result in a z-score comparable to a normal distribution table. RCIs that are > ± 1.64 (90% Confidence Interval, CI) represent meaningful changes, with positive z-scores indicating a significant worsening and negative z-scores indicating a significant improving. Therefore, participants with stable or improved RCI scores on HADS (i.e., equal or lower scores between the 2 time points) at interval 1 (i.e., pre-pandemic to confinement) were classified as resilient individuals (outcome 1), whereas the rest were classified as non-resilient (i.e., significant worsening based on RCI). In addition, four independent and mutually exclusive trajectories were defined considering HADS changes at both analysed intervals (outcome 2): Psychological Resilience (stable or improved at both intervals), Descending (stable or improved at first interval but worsened at second), Recovery (worsened at first interval, improved at second) and Non-resilient (worsened at two intervals).

### Predictive factors

*Lifestyle and psychosocial habits during the COVID‑19 confinement.* We developed an ad hoc questionnaire to assess the main lifestyle and psychosocial habits [27]. As lifestyle factors, leisure and physical activities were computed using two parameters: frequency (scoring range 0–4: “never” to “everyday”) and intensity (scoring 1–4: “No minutes” to “more than 60 minutes”). Moreover, the questionnaire measured psychosocial habits such as number of social interactions -without counting cohabitants- (scoring range 0–5: “None” to “More than 30”), feeling of emotionally supported (yes/no), duration of sleep (5 categories from ‘less than 7 hours’ to ‘more than 12 hours’) and changes in tobacco/alcohol intake at confinement (see Reported Categories/Answers in supplemental information). In this study, levels of leisure and physical activities were computed by multiplying frequency and intensity parameters (range score: 0–16), whereas duration of sleep was dichotomised in two categories (less than 7 hours versus 7 hours or more). Finally, changes of alcohol and tobacco consumption were classified under the categories of “No change,” “Decreased,” or “Increased” (see Table 1s).

*Stress perception and stress resilience.* Participants completed the Perceived Stress Scale (PSS), a measurement of 10 self-reported items of stress perception during the confinement. Scores in the PSS range from 0 to 40, with higher ones reflecting a greater perception of stress [52]. Furthermore, we evaluated the participants’ ability to resist or bounce back from stress using the 6-item Brief Resilience Scale (BRS). Higher scores in BRS indicate higher resilience to stress [53].

*Assessment of stressors.* The questionnaire assessed main sociodemographic characteristics of the sample (age, sex, education, income), including housing features (size in m^2^, open space at home), work situation (actively employed, dismissed, on medical leave, temporary employment regulation-TER), living conditions (living alone, with cohabitants, distribution of household responsibilities, living with dependent persons) and having a caregiving role (excluding being a caregiver for individuals without special needs under the age of 18). Specific pandemic-related stressors (i.e., loss of family members at confinement) were also registered.We estimated a total number of stressors, considering the presence of the following factors (yes = 1, no = 0): (1) Living in a small (less than 46 m^2^) apartment; (2) Absence of an open space at home (confinement); 3). Living alone during confinement; 4). Loss of jobs, TER or sick leave; 5). Reporting being a caregiver; 6). Performing most of the house-keeping tasks (i.e., main responsible) during confinement; 7) Absence of farewell after death to relatives or friends.

*APOE Genotyping*. *APOE* status was determined based on the *APOE* ε4 allele. Participants were classified as *APOE* ε4 carriers or *APOE* ε4 noncarriers.

*COVID-19 Diagnosis*. The diagnosis of infection by COVID-19 was established either with a self-reported test (i.e., serological) or clinically by an expert clinician.

### Statistical analyses

Statistical analyses were performed with SPSS Version 28 (IBM Corp., NY, USA). For the sample description, means and standard deviations (SD) were used for the quantitative variables, while percentages were selected for nominal variables. X^2^ statistical contrasts were applied to analyse if significant differences exist between groups in categorical factors. The analysis of variance (ANOVA), using Bonferroni’s correction in post-hoc contrasts, was used for group comparisons in quantitative variables.

After testing differences between the groups, we performed 2 sets of independent hierarchical logistic regression models (LRM) to predict (1) psychological resilience during the confinement (interval 1: pre-pandemic-confinement) and (2) psychological resilience after 1.5 years follow-up (interval 2: confinement-1.5 years follow up).Thus, the effects of lifestyle (i.e., leisure and physical activities, sleep) and psychosocial habits (i.e., social relationships and emotional support) were tested on binomial hierarchical logistics regression models (LRM) to predict the outcome group (Resilient vs. Non-resilient) at the first interval. The predictive validity of each lifestyle habit was calculated without any adjustment (step 1) and after the inclusion of main covariates (i.e., age, sex, having an open space at home, being the main responsible of house chores or caregiver of dependent persons) that were associated with being resilient at groups comparisons (step 2). Then, the perceived stress was included on each model as final step of the regression models (step 3). Additionally, and following a similar approach, a multinomial LRM was applied to predict Psychological Resilience (reference group) at 1.5 years follow-up versus other psychological courses (Descending, Recovery and Non-resilient) as outcomes. The Hosmer–Lemeshow test was performed to assure the goodness of fit of each logistic model.

As a final stage, different mediation analyses were performed (model 4: bootstrap method) with macro Process (version 4.2 for SPSS) to test the role of perceived stress (mediator variable) on the relationship between lifestyle (e.g., physical activity) or psychosocial factors (e.g., social support) and psychological resilience (i.e., RCI on HADS). The pathways strength was assessed by the standardized regression coefficients (β). An indirect effect was considered significant if the confident interval (CI) did not include zero. The number of bootstrap samples was set at 10,000 and the level of confidence was 95% [54].

## Results

Table 1 shows descriptive characteristics of the four identified groups and the statistical group differences in sociodemographics, living conditions, stressors, clinical and psychological characteristics.

**Table 1:** Characteristics of trajectories groups and statistical comparisons

Variables (*N* = 677)	Psychological Resilience (group 1) *N* = 448	Descending Trajectory (group 2) *N* = 84	Recovery Trajectory (group 3) *N* = 59	Non-resilient (group 4) *N* = 86	F/X^2^	*p*- value and post-hoc
*Sociodemographics*						
Age (years)	63.52 ± 6,34	61.14 ± 6.61	62.71 ± 5.81	62.16 ± 6.64	3.90	<.01^a^
Sex, % men	45.8	33.3	30.5	30.2	13.04	< 0.01
Education (years)	13.94 ± 3.40	13.96 ± 3.37	13.58 ± 3.35	14.01 ± 3.10	0.23	n.s
Income, % medium-high [N=514*]	89.1	81.8	87.5	88.3	2.79	n.s
Work situation (pandemic), % active [*N* = 297*]	71.6	59.5	71.4	61.4	3.51	n.s
*Living Conditions*	45.9	46.3	37.5	45.8	4.10	n.s
Housing size, %high						
Open Space, %yes	94.9	97.4	89.3	89.2	7.99^¥^	< 0.05
Living Alone, %yes	88.7	88.5	78.6	89.2	5.00	n.s
Cohabitants (n); [*N* = 525*]	1.59 ± 1.36	1.85 ± 1.69	1.38 ± 1.30	1.78 ± 1.33	1.34	n.s
Chores, % main charge	26.5	47	48.8	45.8	23.21	< 0.001
Caregivers, % yes	13.5	34.2	5.7	20.3	24.85	< 0.001
Living with, %yes [*N* = 99*]	35.7	54.2	50	53.3	3.12^¥^	n.s
*COVID diagnosis, %yes*	14	12.2	12.2	17.9	1.18	n.s
*APOE 4 carrier, % yes*	36.8	47.0	39.0	43.0	3.74	n.s
*Stress and resilience*						
Total Stressors (n)	2.76 ± 1.05	3.00 ± 1.36	2.67 ± 1.20	2.98 ± 1.13	2.06	n.s
Perceived stress (visit 1) Perceived stress (visit 2)	13.79 ± 7.32 13.79 ± 7.26	18.23 ± 8.25 23.99 ± 9.21	21.53 ± 8.54 16.15 ± 7.65	25.04 ± 8.25 24.66 ± 9.64	59.52 73.43	<.001^a, b,c,*d*,e,*f*^ <.001^a, c,d, f^
Brief Resilience Scale	2.81 ± 0.46	2.82 ± 0.38	2.86 ± 0.39	2.86 ± 0.46	0.48	n.s
*Substance intake*						
Tobacco, % increase [*N* = 69*]	38.1	25	28.6	25	1.12^¥^	n.s
Alcohol, % increase [N=285*]	16.2	27.6	28.6	24.3	4.29	n.s

Note. *Variables with more than 20% of missing values. ^¥^ More than 20% of the boxes showed expected frequencies less than 5. ^ͳ^ ≥1500 euros (per moth); Letters represent significance between pairs of groups (a. Group 1 vs. 2; b. Group 1 vs. 3; c. Group 1 vs. 4; d. Group 2 vs. 3; e. Group 2 vs. 4. f. Group 3 vs. 4) and those in italic a marginal significance *p* <.010. Group (1) Sustained Resilient (stable or improve at both intervals); Group (2) Descending resilient (stable or improve at visit 1 but worsen at visit 2); Group (3) Recovery (worsening at first interval, improve at second); Group (4) Non-resilient (worsening at two intervals). n.s = not significant

The psychological resilience group showed higher proportion of men and reported a more equitable distribution of daily household responsibilities in comparison with other groups. This group also showed a lower proportion of caregivers in comparison with Descending and Non-resilient trajectories. Levels of perceived stress differed between all groups at interval 1, with Psychological Resilience and Descending groups showing the lowest scores. In the second interval, the Psychological Resilience and Recovery groups showed the lowest perceived stress. In terms of perceived stress change, the Resilience Group showed stable mean scores at baseline (visit 1) and 1.5 years follow-up (visit 2), whereas “Descending” (mean score at visit1 < visit 2) and Recovery (mean score at visit1 > visit 2) groups displayed substantial changes (i.e., over 5 points) in perceived stress levels with opposite directions. Perceived stress levels of the Non-Resilient group were the highest and remained stable at both intervals. No differences were found between the groups in terms of number of objective stressors, frequency of *APOE-*ε4 and self-reported COVID-19 infections.

### Group differences in lifestyle and psychosocial factors

The Table 2 depicts the descriptive values and statistical comparisons between the four trajectories groups.

**Table 2:** Differences between trajectories groups in lifestyle and psychosocial factors

Variables (*N* = 677)	Psychological Resilience (group 1) *N* = 448	Descending Trajectory (group 2) *N* = 84	Recovery Trajectory (group 3) *N* = 59	Non-resilient (group 4) *N* = 86	F/X^2^	*p*- value and post-hoc
Physical Activity	8.80 ± 4.68	7.52 ± 4.98	8.22 ± 4.93	6.08 ± 4.87	7.55	< 0.001
Leisure Activities	11.50 ± 4.52	10.16 ± 5.09	10.14 ± 5.12	10.22 ± 5.36	3.40	< 0.05
Social relationships (n)	2.61 ± 1.33	2.62 ± 1.19	2.19 ± 1.31	2.12 ± 1.24	3.49	< 0.05
Feeling supported,% yes	99.0	98.6	88	94.8	25.13^¥^	< 0.001
Sleep, % >7 hs	70.5	66.7	52	50	16.61	< 0.001

Note. More than 20% of the boxes showed expected frequencies less than 5

As shown, the groups differed significantly across all tested lifestyle and psychosocial factors. Thus, the group of Psychological Resilience showed increased physical and leisure activities, as well as comparable or more social contacts than the other groups. Moreover, a higher percentage of participants in the Psychological Resilience group reported feeling emotionally supported and sleeping more than 7 h compared to the other groups.

### Associations between psychosocial and lifestyle factors with perceived stress

Higher scores in physical and leisure activities, sleep, social contacts and emotional support were associated with lower perceived stress at visit 1 and 2. At visit 1, the correlations were as follows: PA (r_xy_=−0.24), leisure activities (r_xy_=−0.20), sleep hours (r_xy_=−0.22), emotional support (r_xy_=−0.21) and social relationships (r_xy_=−0.13). All correlations reached a significance level of *p* <.001. At interval 2, significant correlations between stress and physical activity (r_xy_ =−0.24), emotional support (r_xy_ =−0.18) and sleep hours (r_xy_ =−0.18) were observed at p.<0.001. Leisure activities (r_xy_ =−0.14) and social relationships (r_xy_ =−0.11) were also significant at *p* <.05.

### Prediction of psychological resilience: confinement

The hierarchical binary LRM to predict resilience/non resilience during confinement showed that higher physical activity (Exp(B) = 0.92, 95% CI = 0.88–0.97, *p* <.01), social contacts (Exp(B) = 0.79, 95% CI = 0.65–0.96, *p* <.05) and sleep hours (Exp(B) = 0.42, 95% CI = 0.26–0.66, *p* <.001) were significantly associated with being resilient at confinement (step 1). The effect of the emotional support was on the border of statistical significance (Exp(B) = 0.17, 95% CI = 0.03–1.00, *p* =.05). In the second LRM step, after the adjustment by main covariates (i.e., age, sex, open space, house chores distribution and caregiver role), physical activity (Exp(B) = 0.93, 95% CI = 0.88–0.97, *p* <.01), social contacts (Exp(B) = 0.77, 95% CI = 0.63–0.95, *p* <.05) and sleep duration (Exp(B) = 0.37, 95% CI = 0.23–0.60, *p* <.001) remained significant. Finally, after introducing perceived stress in the LRM (step 3), only social contacts remained significant and sleep hours showed a tendency towards significance (see Table 3).

**Table 3:** Logistic regression models: prediction of mental resilience at confinement

Interval 1: Pre-pandemic to confinement	Wald	Exp (B)	95% CI	*p*
**Model 1** (X^2^ = 65.70, *p* <.001)				
R^2^Nagelkerke = 0.23				
Physical Activity	0.78	0.97	0.92–1.03	n.s
Leisure activities	0.00	1.00	0.94–1.05	n.s
Perceived stress	51.38	1.12	1.09–1.16	< 0.001
**Model 2** (X^2^ = 69.82, *p* <.001)				
R^2^Nagelkerke = 0.26				
Social relationships	3.91	0.79	0.63–0.99	0.04
Emotional support	0.32	0.53	0.06–4.58	n.s
Perceived stress	38.89	1.11	1.08–1.15	< 0.001
**Model 3** (X^2^ = 90.68, *p* <.001)				
R^2^Nagelkerke = 0.27				
Sleep hours (> 7 hs)	3.15	0.62	0.36–1.05	0.07
Perceived stress	48.86	1.12	1.08–1.15	< 0.001

Exp (B) = Odd ratio; CI = Confidence interval

Age, sex, open space, house chores distribution and caregiver role were added as covariates, but any of these covariates was significant at step 3 (data not shown)

### Prediction of psychological resilience: 1.5 years follow-up

A multinomial LRM was carried out to test the associations between lifestyle and psychosocial factors and resilience trajectory (taken as reference group) at interval 2: confinement-1.5 years follow up. The addition of the main predictors into the model (not adjusted) significantly improved the fit between model and data (X^2^ = 42,77, *p* <.001; Nagelkerke *R*^*2*^ = 0.10, *p* <.001). A significant and unique contribution was made by physical activity (X^2^ = 12,86, *p* <.01), social contacts (X^2^ = 8,77, *p* <.05), sleep duration (X^2^ = 8,80 *p* <.05), but leisure activities was not significant (X^2^ = 6,00, *p* =.11). Emotional support was not included in the model since case variability was null in some of the strata. After the introduction of the main covariates- see Table 1 (age, sex, open space, chores distribution and caregiver role) in the second step (X^2^ = 72,77, *p* <.001; Nagelkerke *R*^*2*^ = 0.19, *p* <.001), physical activity (X^2^ = 14,30, *p* <.01) and sleep duration (X^2^ = 8,87, *p* <.05) remained as significant predictive factors, whereas social contacts showed a non-significant trend (X^2^ = 6,83, *p* =.07). Finally, when perceived stress (X^2^ = 68,84, *p* <.001) was added into the model -third step- (X^2^ = 141,62, *p* <.001; Nagelkerke *R*^*2*^ = 0.35, *p* <.001), none of the covariates, lifestyle and psychosocial factors reached the significance threshold. Table 4 depicts the adjusted Multinomial LRM considering the Psychological Resilient group as the reference category.

**Table 4:** Multinomial logistic regression: prediction of resilience at 1.5 years follow-up

	B	Wald	Exp (B)	95% CI	*p*
**Descending Trajectory**					
Physical Activity	−0.07	4.40	0.93	0.87–0.99	p.<05
Caregiver Status	0.37	6.13	0.39	0.19–0.82	*p* <.05
**Recovery Trajectory**					
Chores distribution	−1.00	4.06	0.36	0.13–0.97	< 0.05
**Non-resilient Trajectory**					
Sex	−0.77	4.19	0.45	0.21–0.96	< 0.05
Physical Activity	−0.10	9.72	0.89	0.84–0.96	< 0.01
Social Contacts	−0.27	4.47	0.76	0.58–0.98	< 0.05
Sleep hours	0.92	8.12	2.56	1.34–4.90	< 0.01

The group of sustained/growth resilience (SPR) was considered as reference

As shown, physical activity level, number of social interactions and sleep hours increased the probability of being resilient rather than non-resilient. The effect of physical activity was also significant for the comparison between Psychological Resilient and Descending trajectory groups, reducing the probability of exhibit anxious/depressive symptomatology at the second interval. Further, the absence of caregiving role, an equal distribution of house chores, and men category were associated with higher probability of being resilient against other groups (Descending, Recovery and Non-resilient).

#### Mediation analyses: psychosocial and lifestyle factors, stress and mental resilience

**Fig. 3:**
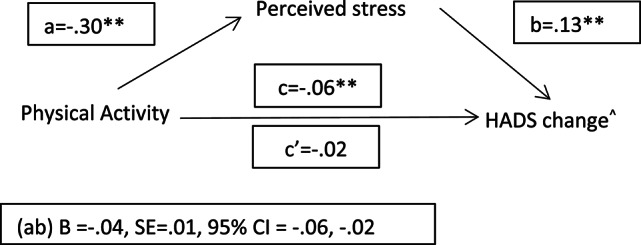
Mediation model of stress on physical activity and mental resilience relationship. Note. ^ Reliable change index of HADS at interval 1 (pre-pandemic-confinement). A positive score indicates a significant worsening. whereas a negative score means a significant improvement. See methods for the details about the reliable change index computation

**Fig. 4:**
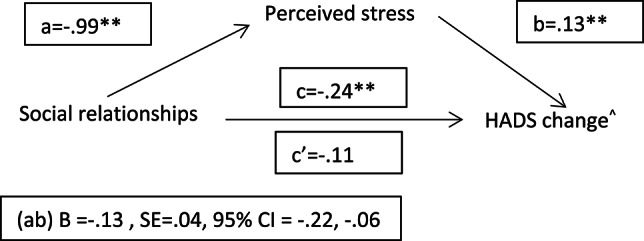
Mediation model of stress on social relationships and mental resilience association. Note. ^ Reliable change index of HADS at interval 1 (pre-pandemic-confinement). A positive score indicates a significant worsening. whereas a negative score means a significant improvement. See methods for the details about the reliable change index computation

**Fig. 5:**
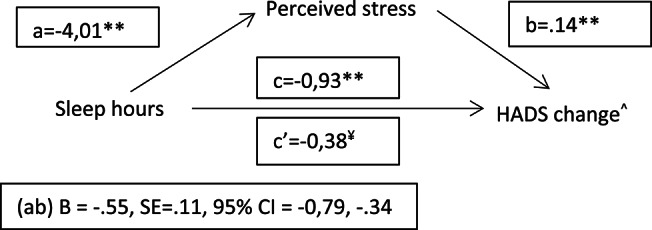
Mediation model of stress on sleep and mental resilience relationship. Note. Marginal significance is shown by ¥. ^ Reliable change index of HADS at interval 1 (pre-pandemic-confinement). A positive score indicates a significant worsening. whereas a negative score means a significant improving. See methods for the details about the reliable change index computation

Based on previous LRMs, we tested whether perceived stress at both intervals mediated the relationship between the psychosocial and lifestyle factors and a continuous measure of psychological resilience (i.e., RCI change on HADS). The results (Figs. 3, 4 and 5) showed indirect effects (ab) of higher levels of physical activity, social relationships and sleep hours on psychological resilience through lower stress perception during confinement. At two years follow-up, partial indirect effects of PA (B=−0.02, SE = 0.01, 95% CI =−0.04, − 0.01), social contact (B=−0.06, SE = 0.03, 95% CI =−0.11, − 0.01) and sleep (B =−0.27, SE = 0.07, 95% CI =−0.42, − 0.13) on psychological resilience through stress perception were also observed (figures not shown).

## Discussion

This longitudinal study investigated the psychological trajectories at two intervals of COVID-19 pandemic in cognitively unimpaired adults at risk of AD. The main findings of our study were as follows: (1) we identified 4 psychological trajectories, with the majority of participants (66,1%) being on the resilience trajectory; (2) resilience trajectories were consistently predicted by higher physical activity level, a greater number of social interactions and longer sleep duration (at least 7 h/night) at confinement and 1.5 years follow-up; (3) these associations were dependent on perceived stress levels, suggesting that stress plays a mediating role in the relationship between lifestyle, psychosocial factors and psychological resilience.

As previously mentioned, four subtypes of psychological trajectories were identified at these intervals. Thus, individuals with Psychological Resilience (i.e., stable or improved at both intervals) were most frequent (66,1%) in our sample. Descending (12,4%) and Non-Resilience trajectories (12,7%) were at similar prevalence rates, whereas those showing Recovery (i.e., worsening at first interval, improve at second) were the most uncommon (8,7%). These findings support previous data indicating that the effect of restrictive measures on mental health symptoms during the pandemic were partially heterogeneous (7). In addition, our observations are in accordance with other studies [26] supporting that older adults (> 70%) usually show a resilient trajectory during the COVID-19 pandemic. However, this study entails a particular interest as anxiety and depression at preclinical AD stages may be correlated with AD biomarkers [11], a finding that may be particularly relevant for individuals with increased AD risk [7].

Overall, our findings elucidate specific modifiable lifestyle and psychosocial factors that may help to prevent mental health problems during stressful situations– such as pandemics - in populations with high risk of AD. In particular, the logistic regression models showed that higher levels of physical activity, social contacts and sleep duration consistently predicted psychological resilience at lockdown and two years later, even after controlling for the effect of different covariates (age, sex, open space, house chores distribution and caregiver role). In line with this, Carriedo et al. (2020) [34] also found a significant relationship between higher physical activity level and lower depressive symptoms in older adults within the COVID-19 restrictions in Spain. Extending these findings, Killgore et al. (2020) [42] also revealed that psychological resilience was greater among adults who tended to exercise more, perceive more social support, sleep more hours, and pray more often during the COVID19 lockdown. In addition, our study showed that the four groups based on HADS reliable change did not differ on the brief stress resilience scale scores. This result was not expected based on previous literature indicating that psychological resources (i.e., self-efficacy, resilience) predict better psychological trajectories [22]. We found almost equivalent scores and standard deviations on this scale in all groups, which may be attributed to either the homogeneity of this construct in this sample or the limited score interval of the measure.

It is important to highlight that no differences were found in total number of stressors, including COVID-19 diagnose, but significant differences in perceived stress emerged between the groups. This observation emphasizes that objective and subjective measures of stress assess different aspects [55], and subjective perception is essential to the psychological impact of a given stressful event or situation [56]. Consistently, the results indicated that perceived stress levels were closely related to changes in HADS scores, with Psychological Resilient group showing the lowest stress levels at both intervals, whereas the Non-resilient group had the highest stress levels. Previous studies indicated that levels of perceived stress are closely related to negative psychological consequences (i.e., anxiety, depression) during the pandemic [57]. In our sample, perceived stress was the unique predictor of psychological trajectory at 1.5 years of follow-up, overshadowing the effects of positive lifestyle habits and psychosocial factors. Hence, a consistent indirect effect of lifestyle habits (physical activity, social contacts and sleep) on psychological resilience was exerted through perceived stress (mediator) at two intervals. Likewise, other studies have pointed out the benefits of higher level of physical activity for psychological distress during the pandemic [58]. These findings are also consistent with the study of Matovic et al. (2023) [41], demonstrating that insomnia problems and loneliness were associated with increased psychological distress during COVID-19 pandemic.

In this study, the reference/normative group (Psychological Resilience) was slightly older and mainly composed by men. Besides, this reference group showed a more equitable distribution of household responsibilities in comparison with the other groups. The presence of an open space at home at confinement was also more frequent in those participants that stayed stable or that improved during the first interval, whereas the caregiver role was less frequent in the psychological resilience and recovery groups. Previous literature has shown that sociodemographic factors, such as younger age and being a woman, are associated with worsening’ mental trajectories during COVID-19 pandemic [8, 16]. Likewise, it is well known that caregivers reported increased stress and associated psychological problems during the pandemic [59], whereas those getting outside more often tended to be more resilient [42]. In contrast, other factors associated with poorer psychological trajectories (lower education, being unemployed, and/or reporting lower income) did not differ between the groups. In this regard, the influence of individual factors on psychological trajectories may also depend on to contextual and social circumstances [60]. For instance, the ALFA is a cohort of relatively highly educated participants, most of them employed during the pandemic, with moderate-high income levels and living usually alone during the lockdown.

The current study has a series of limitations to be considered. Firstly, the sample size is limited and the results may not be generalized to the population at increased AD risk. However, to the best of our knowledge, this is the first study investigating psychological trajectories in CU older adults at increased risk of AD during the COVID-19 pandemic. Secondly, the computation of RCI based on Hsu’s method has some advantages such as the correction of regression to the mean effect, but there is no definitive agreement about the optimal procedure to estimate reliable changes [61]. Finally, data on covid infection were self-reported and missing data were substantial.

Overall, we conclude that cognitively unimpaired individuals of the ALFA cohort maintained, in general, a stable or resilient psychological trajectory during the COVID-19 pandemic. Moreover, lifestyle and psychosocial habits such as physical activity, sleep duration and social support were significantly associated with lower stress and psychological resilience during the pandemic. The mediation effect of perceived stress on the relationships (i.e., lifestyle/psychosocial factors and psychological trajectories) indicates that they exert an indirect impact on mental health by reducing perceived stress. Altogether, the findings underscore the role of physical activity, social interactions and sleep quality to mitigate the impact of stress on mental health and promote psychological resilience during prolonged health crises such as the COVID-19 pandemic. These lifestyle and psychological factors may be valuable targets for public health strategies aimed to prevent mental health problems in older adults who are exposed to high levels of stress.

## Data Availability

All data produced in the present study are available upon reasonable request to the authors.
